# Combined PCL and anatomic posterolateral corner reconstruction: A tibial slope under 8 degrees and a persisting dorsal instability of 4 mm or more have a negative effect on the clinical outcome

**DOI:** 10.1007/s00402-024-05619-5

**Published:** 2024-12-21

**Authors:** H. Fahlbusch, S. Weiß, A. Korthaus, R. Akoto, M. Krause, K. H. Frosch

**Affiliations:** 1 Department of Trauma and Orthopaedic Surgery, University Medical Center Hamburg-Eppendorf, Hamburg, Germany; 2 Department of Trauma and Orthopaedic Surgery, Cologne-Merheim Medical Centre (CMMC), University of Witten/Herdecke, Cologne, Germany; 3 Department of Trauma Surgery, Orthopaedics and Sports Traumatology, BG Klinikum Hamburg, Hamburg, Germany

**Keywords:** Reconstruction, Posterior slope, Posterior tibial translation, Posterior cruciate ligament, PCL, Posterolateral corner, PLC

## Abstract

**Purpose:**

The failure rate following posterolateral corner reconstruction (PLC) remains high. Previous research indicates that in posterior cruciate ligament (PCL) reconstruction the laxity is affected by the tibial slope (TS). However, there is currently no literature evaluating the impact of TS on surgical outcome in combined reconstruction of PLC/PCL.

**Methods:**

This study analyzed 47 patients in a retrospective cohort study who underwent PCL and anatomical PLC reconstruction according to techniques described by Arciero or LaPrade. TS was measured, and patients were divided into two groups: Group A (n = 16) with TS < 8° and Group B (n = 31) with TS ≥ 8°. After a minimum follow-up of 12 months, the side-to-side difference (SSD) of posterior tibial translation (PTT) was assessed using instrumented stability testing (Rolimeter), and various patient-reported outcome measures (IKDC, Lysholm) were collected and compared.

**Results:**

At a mean follow-up of 17.7 ± 4.7 months, group A exhibited a higher SSD of PTT (A 3.9 mm ± 2.1 vs. B: 2.8 mm ± 1.5; p < 0.05). A negative correlation was observed between SSD of PTT and both TS (r = − 0.43; R^2^ = 0.18; p < 0.01) and the Lysholm Score (r = − 0.41; R^2^ = 0.17; p < 0.01) in the overall cohort. Subgroup analysis revealed a higher Lysholm Score in patients with a postoperative SSD of PTT less than 4 mm (85.7 ± 10.1 vs. 79.2 ± 6.6; p = 0.0006).

**Conclusion:**

In combined PCL and anatomic PLC reconstruction a TS < 8° results in elevated SSD of PTT. A flattened TS is linked to higher remaining SSD of PTT, while lower SSD of PTT is associated with improved clinical outcomes.

**Level of evidence:**

Retrospective Cohort Study, IV.

## Introduction

Soft tissue structures like the posterior cruciate ligament (PCL), posterolateral corner (PLC), and meniscus have long been recognized for their roles in posterior knee laxity. The PLC comprises the lateral collateral ligament (LCL) and the popliteus complex, which includes the popliteus muscle tendon unit and the arcuate complex. The arcuate complex consists of the popliteofibular ligament, the fabellofibular ligament, and the popliteomeniscal fibers. The popliteus complex is the crucial stabilizer against external tibial rotation and posterior translation [12, 24]. Arciero’s [3] fibula based and LaPrade’s [23] tibiofibular based anatomical PLC reconstructions are common techniques and proven to restore sufficient stability [38] as well as yielding equally satisfying clinical results [6, 7, 41].

However, the impact of tibial plateau bony geometry, particularly the tibial slope (TS), has remained less explored. Emerging research suggests that the inclination of the tibial plateau directly influences knee kinematics, including anteroposterior laxity, cruciate ligament loading, and the center of rotation [2, 17, 31]. Notably, the importance of the PLC in knee stability has gained prominence. Studies underscore the pivotal role of the PLC in maintaining knee stability, emphasizing its interaction with the TS, indicating that changes in TS can significantly affect load transmission within the knee joint and the anterior–posterior translation of the tibia [1, 16, 29]. An elevated TS, for instance, has been linked to potentially reducing mechanical graft overload following PCL reconstruction [19]. Conversely, a flattened TS is associated with markedly increased posterior tibial translation (PTT), providing insights into why existing surgical approaches struggle to restore knee stability effectively [17]. Despite these insights, there remains a gap in our understanding of how the TS influences posterior laxity, particularly in the context of anatomic posterolateral reconstruction (Arciero or LaPrade).

To the best of our knowledge, this study is the first to investigate the relationship between TS and PTT in the anatomical reconstruction of PLC/PCL deficient knees. Our primary objective was to explore whether a correlation exists between TS and posterior laxity following PLC and PCL reconstruction, with a specific focus on the importance of anatomic PLC reconstruction in knee stability. We hypothesised that elevated TS will correspond to a greater reduction in PTT following PLC and PCL reconstruction.

## Materials and methods

**Fig. 1:**
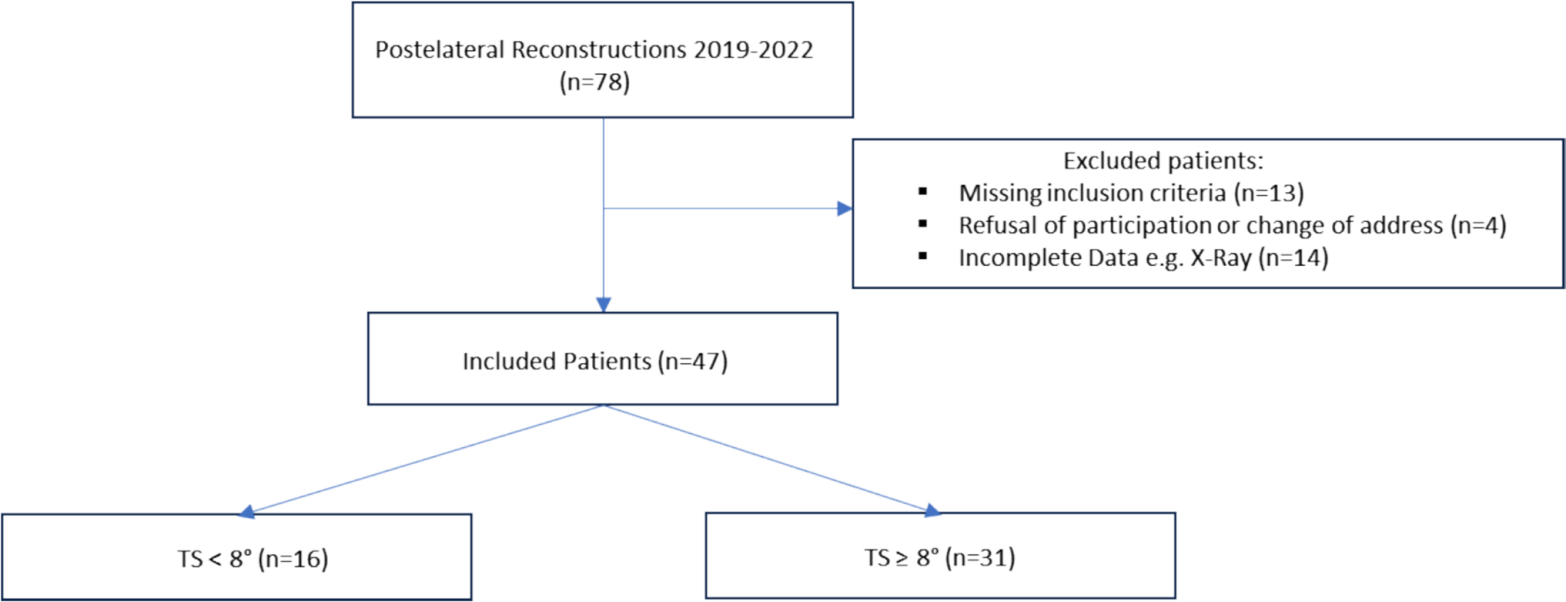
Flow Chart of patient selection

Patient population: this study used a retrospective cohort study design. The in-house trauma register was screened at two level I trauma centres for all patients undergoing combined PLC and PCL reconstruction surgery between 2018 and 2022 (see Fig. 1). Only patients presenting with chronic injuries (> 6 weeks), a combination of varus and posterolateral instability (Fanelli B [14], see Table 1) and additional posterior instability (> 5 mm of side-to-side difference on bilateral posterior stress radiographs, Telos) due to PCL injury were included. Diagnosis was based on MRI-imaging, stress radiography and physical examination to assess ligamentous instability. Exclusion criteria were patient age under 18 years, higher grade cartilage defects (ICRS > 2), peroneal nerve injuries, higher- or lower-grade PLC injuries (Fanelli A/C), revision PCL/PLC reconstruction, missing radiographic data, varus or valgus malalignment as determined by long leg radiographs, prior slope correcting osteotomy, and additional affected structures (e.g. ACL, MCL). All included Patients were divided into two groups according to their TS based on the findings of Gwinner et al. [17]: group A with TS < 8° and group B with TS ≥ 8°. An institutional ethics commission approved this study and informed consent was obtained by each patient prior to clinical follow-up investigation.

**Table 1:** The Fanelli and Larson classification [14] classifies the damage in posterolateral structures

Fanelli and Larson classification	Scale of damage
Type A	> 10° Increase in External Rotation (ER) of Tibia
Type B	> 10° increase in ER of Tibia + mild-moderate instability in varus stress
Type C	> 10° increase in ER of Tibia + severe instability in varus stress

Surgical management: All PCL reconstructions were arthroscopically performed by a single bundle technique. Hamstring tendon autografts were used in all cases. Additional posterolateral reconstruction according to Arciero [3] or LaPrade [23] or their derivative arthroscopic reconstructions [15, 22] were performed. The choice of surgical technique was initially randomised, but later changed in favour of Arciero’s technique, as the clinical outcomes are equivalent [13, 41], but Arciero’s technique was simpler and less invasive. Arciero’s technique utilizes a fibula-based reconstruction with an anatomic transfibular tunnel placement along the native functional anatomy of the LCL and popliteus tendon. In contrast, LaPrade’s approach includes an additional tibiofibular graft to address tibiofibular instability.

Rehabilitation: peripheral nerve block anaesthesia was uniformly administered. Standardized physical therapy commenced 48 h post-operation, incorporating 6–8 weeks of wearing stabilizing braces (Jack PCL Brace; Albrecht, Bernau am Chiemsee, Germany). The initial 6 weeks involved a restricted range of motion, with a maximum knee flexion of 45° for the first 2 weeks. Passive knee flexion was initiated after drain removal, followed by progressive mobilization to 60° up to week 4, and 90° in weeks 5 and 6. Weight-bearing was limited to 20 kg for six weeks, transitioning to full weight-bearing after this period.

**Fig. 2:**
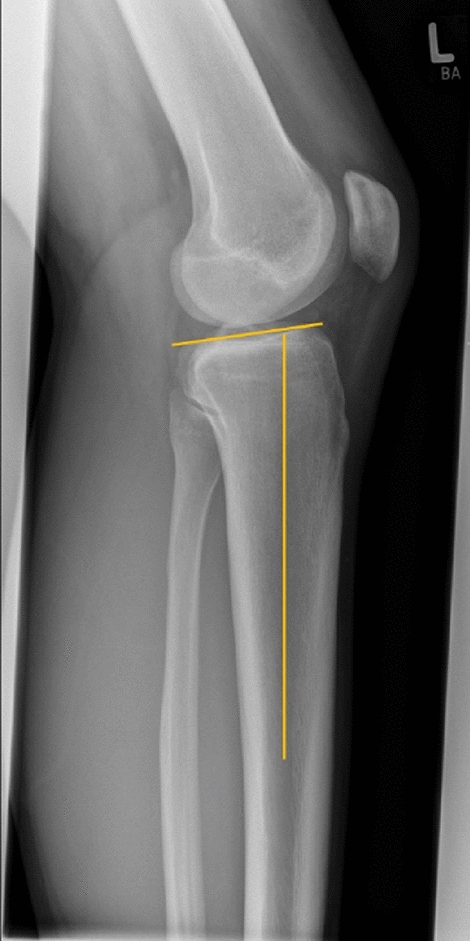
Preoperative measurement of the Tibial Slope (TS): The TS is defined as the angle between a line parallel to the posterior inclination of the tibial plateau and a line perpendicular to the diaphyseal shaft axis

Clinical testing and radiological assessment: two independent observers (A.K. and H.F.) assessed the TS and PTT, with both parameters measured in a blinded fashion on preoperatively performed bilateral Telos Stress Radiographs. Mean values for the TS and PTT were subsequently computed. TS, defined as the angle between a line parallel to the posterior inclination of the tibial plateau and a line perpendicular to the diaphyseal shaft axis, was measured following the method by Dejour et al. [11]. The medial TS was selected due to the superimposition of the medial contour on lateral radiographs and recent literature suggests that it might be more significant than the lateral TS in PCL injuries [25]. The diaphyseal shaft axis was established using midpoints between the anterior and posterior tibial cortex at specified locations (9 and 15 cm distally to the joint line). The tangent line to the medial tibial plateau and the perpendicular to the diaphyseal axis determined the TS. In cases of multiple radiographs, TS was measured on the one containing the longest diaphyseal axis and the best true lateral view. (see Fig. 2).

A follow-up examination, conducted at a minimum of 12 months post-surgery, utilized Lysholm and IKDC scoring systems for functional outcomes. The posterior drawer test was performed on both knees using the Rolimeter test to assess PTT according to Höher et al. [22], and from this data, the postoperative side-to-side difference of the PTT (SSD of PTT) was calculated.

A cut-off value of 4 mm SSD of PTT was chosen to divide patients into clinically significant instability [32, 43]. Postoperative PCL graft failure was defined as non-traumatic PCL re-rupture confirmed by MRI or arthroscopy.

### Statistical analysis

Statistical analysis was performed using GraphPad Prism 8 (San Diego, CA, USA). Data are presented as means and standard deviations (SD). The calculation was based on two groups: (1) patients with a TS < 8° (Group A) compared to (2) patients with a TS ≥ 8° (Group B). The intra-rater and inter-rater reliability for the measurement of the tibial slope (TS) were evaluated using the intraclass correlation coefficient (ICC). The primary outcome was defined by instrumental stability testing (SSD of PTT) and the secondary outcome by PROMS (Lysholm and IKDC-Score). Additional subgrouping was performed for BMI (Body mass index), surgical technique and SSD of PTT. The differences between the groups were calculated using the student’s t-test and Mann Whitney U-Test or Wilcoxon matched-pairs rank test for non-parametric parameters. Categorical parameters were compared using Fisher’s exact test. Pearson correlation analysis examined the association between TS and PTT or PROMS. For group comparisons of categorical variables, the chi-square test was applied. The statistical significance level was set at p < 0.05. Sample size calculation using G-Power (version 3.1.9.7., Heinrich Heine University, Düsseldorf) indicated that a sample size of n = 12 in each group would be required to detect a difference of 10 points with an SD of 10 in the clinical Lysholm score, with a α error of 5% and a power of 0.8. A significance level of p < 0.05 was applied.

## Results

### Patient demographics

Ultimately, 47 patients were included, with a mean follow-up of 17.7 ± 4.7 months (range 12–22): 16 in group A (TS < 8°) and 31 in group B (TS ≥ 8°). The average age was 37 ± 12.0 years (range: 19–61) and time to surgery was 109.1 ± 12.5 weeks (range: 8–526). There were no differences regarding demographic data between groups. One patient (group B) had a tear of the posterior horn of the lateral meniscus, which was successfully resolved by all inside sutures.

### Tibial slope

**Fig. 3:**
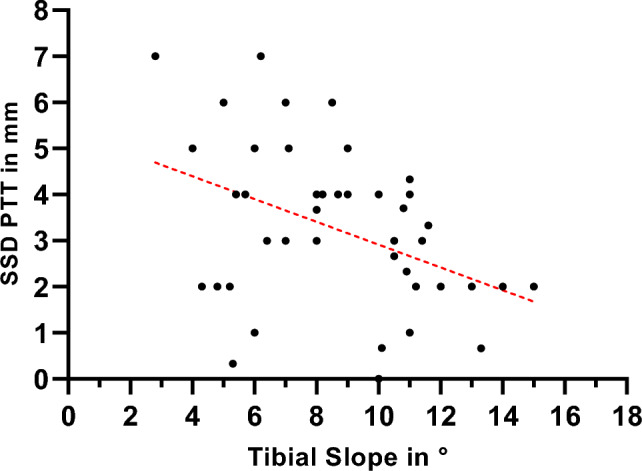
Regression Plot showing a negative association between postoperative SSD of PTT and TS. The central dotted line is the calculated regression line, indicating a significant correlation between TS and SSD of PTT (r = -0.43 and R^2^ = 0.18; p = 0.0037). SSD of PTT Side- to-side difference of posterior tibial translation

The mean TS measured 8.6° ± 3.0° (range 2–15°) for the surgically treated knee and 9.5° ± 3.0° (range 4–16°) for the contralateral knee. Pearson correlation analysis showed a significant correlation between the postoperative SSD of PTT and TS in the overall patient cohort (p = 0.0037) with r = -0.43 and R^2^ = 0.18 (see Fig. 3). However, there was no direct correlation between TS and the clinical outcome (Lysholm and IKDC-Score). The ICC demonstrated strong agreement for both intra-observer reliability (0.93, 95% CI: 0.887–0.938) and inter-observer reliability (0.86, 95% CI: 0.810–0.890) in the measurement of the TS.

### Patient reported functional outcome

The Lysholm Score averaged at 83.0 ± 9.3 and IKDC Score at 73.5 ± 14.5 points at follow-up and showed no difference between groups (see Table 2).

**Table 2:** The Table presents demographics, clinical outcomes and complications categorized by TS

Characteristics	Total (n = 47)	TS < 8° (n = 16)	TS ≥ 8° (n = 31)	p-value
Female sex^b^	11 (23.4)	2 (12.5)	9 (29.0)	n.s
Age in years^a^	37.0 ± 12.0	39.2 ± 10.9	36.2 ± 13.2	n.s
BMI in kg/m^2a^	26.06 ± 3.9	26.3 ± 3.9	26.2 ± 4.0	n.s
Time to surgery in weeks^a^	109.1 ± 12.5	106.8 ± 11.2	110.4 ± 12.9	n.s
Meniscal tear^b^	1 (2.1)	0	1 (3.2)	n.s
Arciero^b^	36 (78.7)	13 (81.3)	23 (74.2)	n.s
Follow-up, in months^a^	17.7 ± 4.7	17.6 ± 4.9	16.9 ± 4.5	n.s
PROMS^a^				
▪ Lysholm Score	83.0 ± 9.3	82.7 ± 8.7	83.2 ± 8.9	n.s
▪ IKDC Score	73.5 ± 14.5	75.3 ± 13.4	70.1 ± 16.1	n.s
SSD of PTT in mm^a^	3.2 ± 1.7	3.9 ± 2.1	2.8 ± 1.5	**0.0457**
Graft failure^b^	1 (2.1)	1 (6.3)	0	n.s
Joint stiffness^b^	1 (2.1)	0	1 (3.2)	n.s

Group A (TS < 8°) had a significantly higher SSD of PTT (3.9 mm ± 2.1 vs. 2.8 mm ± 1.5, p = 0.0457

^a^Mean ± SD

^b^n (in %); SSD of PTT Side- to-side difference of posterior tibial translation; TS Tibial Slope; Bold text indicating p < 0.05

### Posterior tibial translation

**Fig. 4:**
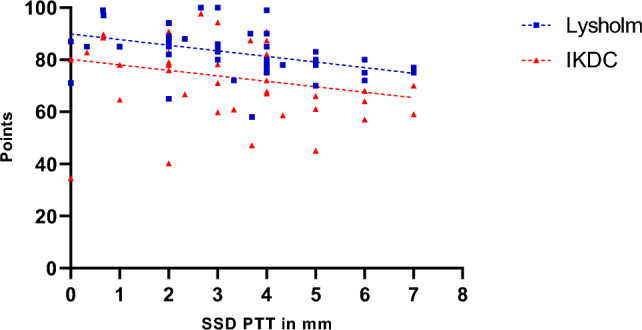
Regression Plot showing a negative association between SSD PTT and Lysholm Score (r = -0.41 and R^2^ = 0.17; p = 0.0048) but not IKDC-Score (r = -0.26 and R^2^ = 0.65; p = 0.0858). SSD of PTT Side-to-side difference of posterior tibial translation

The mean postoperative SSD of PTT was calculated as 3.2 mm ± 1.7 (range 0–7). Group A (TS < 8°), exhibited a significantly higher SSD of PTT in comparison to Group B (TS ≥ 8°): Group A: 3.9 mm ± 2.1 vs. Group B: 2.8 mm ± 1.5; p = 0.0457 (see Table 2). A negative correlation between SSD of PPT and Lysholm Score (r = -0.41 and R^2^ = 0.17; p = 0.0048), but not IKDC Score was shown (see Fig. 4).

### Subgroups

The Lysholm Score and IKDC Score were significantly higher in patients with postoperative SSD of PTT < 4 mm and BMI < 30 kg/m^2^ compared to those with postoperative SSD of PTT ≥ 4 mm and BMI ≥ 30 kg/m^2^, respectively (Lysholm: SSD of PTT < 4 mm vs. SSD of PTT ≥ 4 mm, p = 0.0006; IKDC: BMI < 30 kg/m^2^ vs. BMI ≥ 30 kg/m^2^, p = 0.0484). If postoperative SSD of PTT was ≥ 4 mm, no patient achieved a Lysholm or IKDC Score ≥ 90 points. There were no differences in PROMs between different surgical techniques (Arciero vs. LaPrade). (See Table 3).

**Table 3:** Table showing Lysholm and IKDC Scores divided into subgroups

Subgroups	n	Lysholm^a^	p-value	IKDC^a^	p-value
SSD of PTT < 4 mm	27	85.7 ± 10.1	**0.0006**	76.0 ± 16.0	n.s
SSD of PTT ≥ 4 mm	20	79.2 ± 6.6		69.9 ± 11.4	
BMI < 30 kg/m^2^	38	81.1 ± 7.3	n.s	75.1 ± 13.5	**0.0484**
BMI ≥ 30 kg/m^2^	9	83.5 ± 9.7		75.1 ± 13.5	
Arciero	36	82.5 ± 9.1	n.s	72.7 ± 14.6	n.s
LaPrade	11	84.9 ± 10.2		75.8 ± 14.3	

The Lysholm Score was significantly higher in patients with postoperative SSD of PTT < 4 mm (85.7 ± 10.1 vs. 79.2 ± 6.6, p = 0.0006), while the IKDC Score was higher in patients with a BMI < 30 kg/m^2^ (75.1 ± 13.5 vs. 75.1 ± 13.5, p = 0.0484)

^a^Mean ± SD; Bold text indicating p < 0.05; SSD of PTT Side-to-side difference of posterior tibial translation

### Complications

No patients experienced complications such as vascular or nerve injury, compartment syndrome, deep vein thrombosis, or infection. Only one patient reported joint stiffness (range of motion 0–5–110°), which was resolved by arthroscopic arthrolysis after 46 weeks (range of motion at last follow-up 0–0–125°). In another case, a dislocated femoral PCL flipbutton was noticed during postoperative X-Ray and required revision surgery.

PCL-graft failure occurred in one patient with a TS < 8° (see Table 2), who experienced a non-traumatic re-rupture and required revision PCL reconstruction. The patient's SSD of PTT was 5 mm during the final follow-up.

## Discussion

The main finding of this retrospective study on chronic Fanelli type B combined with PCL injuries is that a TS of less than 8° is associated with higher rates of persisting posterior instability after combined anatomic PCL and PLC reconstruction. Additionally, a flattened TS is linked to increased postoperative tibial translation (SSD of PTT) which ultimately leads to worse clinical outcome (Lysholm-Score).

This study is the first to suggest a potential correlation between TS and PTT in patients undergoing PCL reconstruction concurrently with anatomic posterolateral reconstruction based on a defined preoperative classification according to Fanelli. The findings indicate that an elevated TS results in diminished in situ forces acting on the PCL, aligning with previously documented outcomes [17, 31]. The direct association between TS and tibial translation during weightbearing has been established in both cadaveric models [34, 37] and clinical investigations [5, 11, 17]. The knee’s geometric attributes have a significant impact on clinical outcomes. This is demonstrated by the connection between a flattened TS and increased residual PTT [31, 42]. This association remains consistent even after PCL and PLC reconstruction, leading to a reduced reduction in PTT. Importantly, neither surgical technique nor BMI demonstrated a significant impact on the extent of SSD of PTT, which emphasizes the influence of TS on postoperative knee stability. Furthermore, we found a negative correlation between SSD of PTT and the Lysholm Score, suggesting improved clinical outcomes in stable knees. Specifically, patients with a noteworthy SSD of PTT of < 4 mm and a BMI below 30 kg/m^2^ exhibited significantly higher Lysholm and IKDC Scores, respectively. These findings are consistent with previous research examining patient-reported outcome measures and the restoration of stability, confirming positive outcomes [26, 33, 39, 41]. However, to the best of our knowledge, a cut of value of 4 mm SSD of PTT has never been described in PCL/PLC-deficient knees and needs to be re-evaluated in future research.

Complete restoration of knee kinematics in PLC reconstruction remains elusive, contributing to the failure of a significant number of patients to achieve full recovery [27, 29]. Previous research has primarily focused on PCL/ PLC reconstruction techniques and improper tunnel placement [21, 28, 29]. Building upon previous evidence linking ACL graft failure to TS, as reported by Salmon et al., who documented an 11-fold higher rate of ACL graft failure in patients with a TS of ≥ 12° [8, 30]. This finding aligns with the observations of Gwinner et al. [17], who conducted a subgroup analysis of the patient cohort based on their TS. Notably, they demonstrated a significantly lower reduction in PTT in patients with TS ranging from 5 to 8° compared to those with TS exceeding 8°, with extensive load leading to graft failure. The study by Gwinner et al. [17] has some limitations. These include the use of a non-anatomic PLC reconstruction technique (according to Larson et al. [35]), a lack of preoperative classification of instability, absence of clinical scores, and a mean postoperative SSD of PTT of 4.8 ± 3.3 mm at 12 months and 5.4 ± 3.4 mm at final follow-up, indicating a high failure rate according to our criteria (> 6 mm SSD of PTT). Non-anatomic PLC reconstruction techniques may have a greater influence on the clinical result and remaining postoperative instability compared to more anatomic techniques. However, it is worth noting that Gwinner et al. [17] conducted a long-term observation, which is a strength of their study and a limitations of our presented data. A recent study on patients with PCL reconstruction found that a higher tibial slope (TS) was protective against a positive posterior drawer test, which is also consistent with our findings [9]. The average TS in patients with a positive drawer test was 6.2°, while those with a negative drawer test had an average TS of 8.3°. However, they did not find a correlation between TS and Lysholm or IKDC scores, nor with graft failure.

In recent decades, there has been a growing emphasis on correcting osseous malalignment, extending beyond the coronal plane to address sagittal plane issues, particularly in the context of chronic PCL/PLC instability. An elevated TS can offer protection to the grafts used for combined PCL/PLC reconstruction. While no standardized threshold exists for the degree of slope necessitating concurrent osteotomy, literature indicates potential benefits for patients with flat preoperative TS undergoing slope-increasing osteotomy. Bernhardson et al. [4] found a mean TS of 5.7 in PCL-injured patients compared to 8.6 in those without PCL injury. Weiler et al. [40], studying six patients with chronic PCL deficiency undergoing anterior opening wedge high tibial osteotomy, observed a mean preoperative TS of 3.7°, rising to 11.5° postoperatively. As a result, Kanakamedala et al. suggested considering slope-increasing osteotomy for primary PCL reconstruction with TS less than 5° and, in revision scenarios, for TS less than 7° [20]. Our study, constrained by sample size and ROC analysis limitations, did not pinpoint a specific threshold value.

### Limitations

Conclusions drawn from this study are constrained by the small number of cases, a consequence of the rare occurrence of these complex injuries. While precise inclusion and exclusion criteria were employed, there was variability in the time between injury and surgery, albeit consistently exceeding 6 weeks, defining them as chronic injuries. Clinical follow-up utilized Rolimeter instead of stress radiographs. TS quantification relied on lateral views from standard radiographs, potentially overlooking asymmetries in the tibial plateau facets, as well as chondral or meniscal surfaces [10, 36]. The rarity of PLC injuries precluded matched-pair analysis. Long-term follow-up is essential to substantiate the feasibility of arthroscopic PLC reconstruction, given recent promising results.

## Conclusion

In this scoping study, a tibial slope of less than 8° is associated with higher rates of posterior tibial translation in combined PLC and PCL reconstruction. The tibial slope was negatively correlated with posterior tibial translation, emphasizing the importance of bony alignment for stability. Patients with a SSD of PTT < 4 mm showed better Lysholm Scores, underlining the effect of stability on clinical outcome. The evidence provided suggests that the tibial slope should be considered in the decision-making process for surgeons, particularly in revision surgery. Further studies are needed to determine specific threshold values for slope correction osteotomy in PLC/PCL deficient knees.

## Data Availability

The data that support the findings of this study are available upon reasonable request from the corresponding author. The data are not publicly available due to privacy or ethical restrictions.

## Abbreviations

ACL: Anterior cruciate ligament
BMI: Body mass index
IKDC: International Knee Documentation Committee
ICRS: International Cartilage Repair Society
ICC: Intraclass correlation coefficient
LCL: Lateral collateral ligament
MCL: Medial collateral ligament
PCL: Posterior cruciate ligament
PLC: Posterolateral corner
PROMs: Patient-reported outcome measures
PTT: Posterior tibial translation
SD: Standard deviations
SSD: Side-to-side difference
TS: Tibial slope
VAS: Visual analog scale
